# Graphene Quantum Dots by Eco-Friendly Green Synthesis for Electrochemical Sensing: Recent Advances and Future Perspectives

**DOI:** 10.3390/nano11051120

**Published:** 2021-04-26

**Authors:** Viviana Bressi, Angelo Ferlazzo, Daniela Iannazzo, Claudia Espro

**Affiliations:** Dipartimento di Ingegneria, Università di Messina, Contrada di Dio, Vill. S. Agata, I-98166 Messina, Italy; viviana.bressi@unime.it (V.B.); angelo.ferlazzo@unime.it (A.F.); diannazzo@unime.it (D.I.)

**Keywords:** graphene quantum dots, electrochemical sensors, biomass, green synthesis

## Abstract

The continuous decrease in the availability of fossil resources, along with an evident energy crisis, and the growing environmental impact due to their use, has pushed scientific research towards the development of innovative strategies and green routes for the use of renewable resources, not only in the field of energy production but also for the production of novel advanced materials and platform molecules for the modern chemical industry. A new class of promising carbon nanomaterials, especially graphene quantum dots (GQDs), due to their exceptional chemical-physical features, have been studied in many applications, such as biosensors, solar cells, electrochemical devices, optical sensors, and rechargeable batteries. Therefore, this review focuses on recent results in GQDs synthesis by green, easy, and low-cost synthetic processes from eco-friendly raw materials and biomass-waste. Significant advances in recent years on promising recent applications in the field of electrochemical sensors, have also been discussed. Finally, challenges and future perspectives with possible research directions in the topic are briefly summarized.

## 1. Introduction

Over the last thirty years, both academic and industrial chemical research has increasingly oriented towards a holistic vision focused on pollution, use of renewable sources, and waste reduction, leading to the generation of a new concept of chemistry, called Green Chemistry, which with its 12 principles aims to redirect the chemical industry along paths of eco-sustainability. Indeed, sustainable development, which has become increasingly central to scientific and technological progress in the last century, requires chemistry to play a primary role in the conversion of old technologies into new “clean” processes and in the design of new products and new processes that are more eco-friendly, breaking the old paradigms based on the generation of large amounts of waste and the wide use of petrochemicals. One of the most important goals of green chemistry and resource efficiency, as stated in the seventh principle, is the design and development of synthetic approaches with low environmental impact, without the use of harmful solvents. On this account, renewable feedstocks, such as biomasses, constituted of a multifaceted array of low and high molecular weight products, such as sugars, hydroxy and amino acids, and biopolymers such as cellulose, hemicelluloses, or other raw materials easily obtainable from natural sources represent the right direction for sustainable production of fuels and novel advanced functional materials, as opposed to unsustainable production from non-renewable fossil resources such as oil, coal and natural gas [1]. 

Among advanced functional materials, carbon, one of the most abundant elements in the biosphere, plays a crucial role in the development of high-performance and sustainable materials. It is well known that carbon-based materials comprise the most effective properties among all the resources on the earth, such as light weight, high porosity, high-temperature resistance, acid and alkali resistance, good structural stability, and easy conductivity. The above-mentioned characteristics, together with the small background current, the wide potential window, and good electro-catalytic performance have made carbon materials effective in many applications and devices with unlimited possibilities for development [2]. 

GQDs are newly emerging members of the carbon materials family. GQDs are small fragments of graphene with lateral dimensions less than 100 nm, with properties deriving from both graphene and carbon points [3]. In addition to biocompatibility [4] and low toxicity [5,6]. GQDs have characteristics that make them ideal candidates for use in various fields. The high surface area and abundance of functional groups, as well as their easy functionalization with organic, inorganic, or biological molecules [7], has led to the use of GQDs as electrode modifiers. Moreover, they are chemically stable, water-soluble, robust, inert, and photo-stable against blinking and photo-bleaching [8]. Their solubility in water-based solvents has influenced their application in the field of bio-imaging [9,10] and targeted drug delivery [11]. GQDs exhibit attractive optical absorption properties with a peak between 260 and 380 nm making them ideal candidates for the fabrication of photodetectors or optoelectronic devices [3,12]. Another important feature is the excellent photoluminescence property (PL): normally, the quantum yield PL (QY) is high thanks to the crystallinity and the presence of layers in the structure of GQDs. GQDs have high-speed electron transport due to quantum confinement and an edge effect that directly affect electrical conductivity [13,14]. GQDs can act as a good sensing material due to their high electron movement with a high-speed reaction, making them excellent candidates for sensing applications. Moreover, GQDs possess peroxidase mimetic activity originating from their aromatic structure and this explains the strong interest in the development of electrocatalytic H_2_O_2_ detectors [15]. Therefore, taking into account the above mentioned electrochemical properties, great research interest in the use of GQDs for the design of novel electrode materials, not only in the field of fuel cells [16], supercapacitors [17] and photovoltaic cells [18], but also in the field of electrochemical immunosensors for biomedical applications [8] and biosensors [19], has been recently shown. As well as other carbonaceous materials, GQDs are conventionally synthesized from fossil feedstocks such as oil, coal, and petroleum coke and often require energy-intensive synthetic routes and severe process conditions [20]. On the contrary, biomasses or their constituents, such as carbohydrate or organic acids, characterized by their high availability, biodegradability, and low cost, are the only renewable carbon sources and crucial precursors of carbonaceous materials. Moreover, although to date only a few data are available regarding the costs of producing GQDs from renewable precursors, they are expected to have a much lower economic impact than conventional feedstocks (CNts, graphite, etc.), since the various functional groups already existing in the structure of biomass makes the fragmentation easier, related to the dense well-ordered single component graphene or CNTs. On the other hand, the conventional management of biomass waste involves noticeable economic and environmental problems, since traditional disposal strategies, such as incineration or landfilling, are insufficient in terms of environmental impacts, human health, and energy efficiency [21]. Indeed, the development of green routes to obtain GQDs, derived not only from renewable resources, such as lignocellulosic biomass waste, but also from other natural products present in food/agricultural waste (i.e., carbohydrates, lignin, proteins, etc.), not in competition with food suppliers, and without the use of any passivating, reducing, oxidizing agents or organic solvents, is a hot research topic of the 21st century. 

On this account, this review aims to introduce the reader to the latest advances, over the past five years, in green approaches, based not only on the use of biomass wastes, but also on the conversion of natural inexpensive organic molecules, such as glucose or citric acid, simply extracted from a variety of fruits and vegetables, for the synthesis of GQDs, via processes meeting the requirements of the principles of Green Chemistry, and discovering their promising recent applications in the field of electrochemical sensors, to reach the two fundamental goals of meeting increasing energy demand and renewable feedstocks recycling and exploitation.

## 2. GQDs from Eco-Friendly Raw Materials by Green Approaches

The synthesis methods of GQDs are generally classified into two groups, based on the reaction mechanism involved: top-down and bottom-up. The top-down approach consists of cutting down large graphene sheets, carbon nanotubes, carbon fibers, or graphite into small pieces of graphene sheet. Since top-down processes involve the conversion of macromolecules using physical forces into smaller ones, the main reaction mechanism involved is oxidative cleavage, although hydrothermal process is preferred, because it is simpler and faster than oxidative methods. Other top-down processes are electrochemical oxidation, microwave irradiation, and laser ablation. The strategy of the bottom–up method is the use of small molecules as starting materials for the production of the GQDs [22]. The bottom-up technique consists of the controllable synthesis of carbon sp^2^ from organic polymers, or of pyrolysis/carbonization processes starting from organic molecules. Typically, polycyclic aromatic hydrocarbon molecules are the most reliable precursors to form high-quality GQDs [23]. The first method results in a complicated process but has the advantage of obtaining products that can be controlled in terms of size and morphology. Carbonization, on the other hand, is an ecological, easy method, but the structure and the morphology of the GQDs are not controllable and the yield is lower. However, both top-down and bottom-up methods involve the use of very expensive non-renewable raw materials, such as CNTs, graphene, graphene oxide, or other graphene-based precursors, which, if prepared from bulk graphite, require the use of toxic chemicals and strong acidic treatment for the disintegration of the strong and well-ordered structure of graphene into small-sized GQDs, as well as high pressure, high-temperature equipment, resulting in low yields and limited production scalability [24].

The use of green synthetic routes is an emerging area in the field of nanotechnology and offers economic and environmental benefits as an alternative to conventional methods. As is well known, the preparation of graphene quantum dots often needs strong acids or organic solvents, and their green production via sustainable strategies, involving non-toxic and biosafe reagents, still faces important challenges; therefore, eco-friendly synthetic approaches, with easy separation and without complicated post-processes, should be designed and developed.

**Figure 1:**
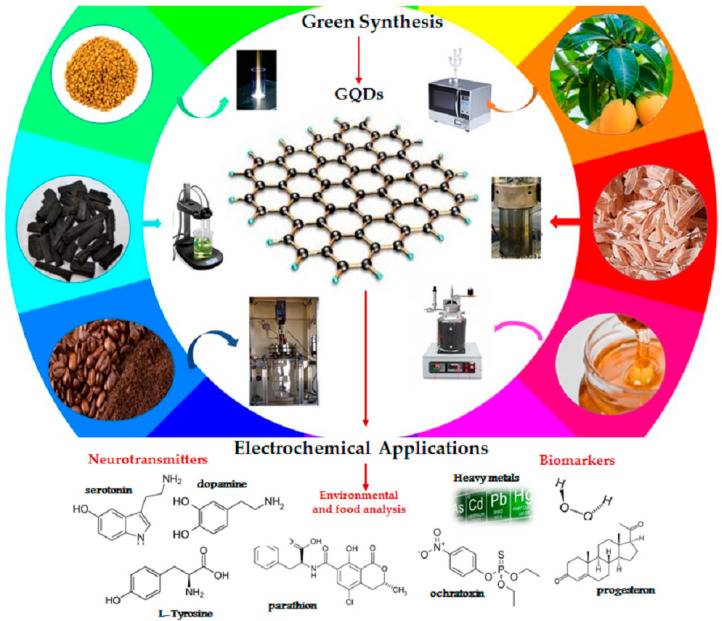
Green synthesis of GQDs and their recent applications in electrochemical sensors [9,27,28,31,32,33,34].

The synthesis of carbonaceous nanomaterials and the choice of precursor materials can be considered equally relevant as, depending on the process and the feedstock, the product will have different features affecting its future applicability. Furthermore, yields that guarantee large-scale production also depend on them. As mentioned in the introduction, in recent years interest has been growing in exploiting adequate renewable resources to decrease dependence on non-renewable resources and increase energy security and environmental safety [25], leading to the development of several attempts to exploit different natural carbon sources for the production of GQDs, by combining both top-down and bottom-up processes. The real advantage of bio based GQDs is the possibility of using a wide range of precursors and several technological approaches [26]. Several biomaterials have been already proposed for a wide range of electrochemical applications to obtain biomass-derived GQDs, ranging from simple and natural molecules to complex compounds, including wheat straw, wood charcoal, rice husk, coffee ground, forestry processing residue, livestock and poultry manure, organic waste from food processing, and municipal solid waste (Figure 1) [9,27,28]. Among these, citric acid (CA), a weak organic acid, and glucose, a carbohydrate, both available in nature, are undoubtedly the most popular carbon precursors, because of their biocompatibility, low cost and ease of supply. Furthermore, carbonaceous materials obtained via green synthesis from CA and glucose, show both photoluminescence from blue to red regions [29] and extremely high QYs (more than 80%) [30].

In the following paragraphs, a selection of the main innovative and interesting synthetic approaches proposed in the last years, as summarized in Table 1, involving the use of biomass waste or other natural starting materials, by green routes for the production of graphene quantum dots, focusing attention on their eco-compatibility and their perspectives for future development, will be discussed.

### 2.1. Oxidative Method 

**Figure 2:**
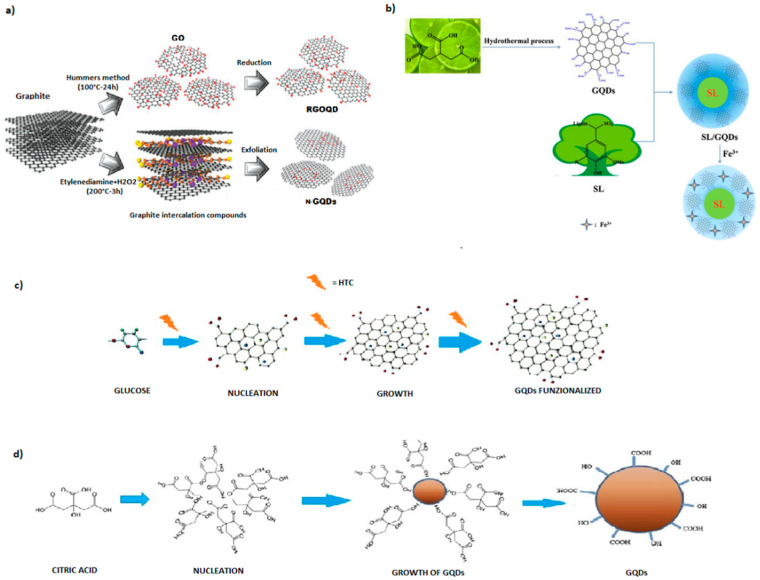
Four green approaches adopted to prepare GQDs. (**a**) Illustration of the preparative strategy for N-GQDs by electrochemical exfoliation and RGOQDs from GO with Hummers method. (Adapted with the permission of Ref. [46]). (**b**) Synthetic one-step process for the production of SL/GQDs used as sensing platform for Fe3+ detection by Xu et al. (Adapted with the permission of Ref. [64]). (**c**) The hydrothermal strategy for the functionalization of GQDs synthetized from glucose. (**d**) Schematic representation of the nucleation and growth of GQDs obtained from citric acid.

Oxidative cleavage is one of the most versatile approaches frequently used for the synthesis of GQDs from larger graphitized carbon materials with relatively high yields. An interesting class of nearly uniform size (~5 nm) of graphene quantum dots (E-GQDs) was prepared via eco-friendly carbon electro-oxidation of wood charcoal by Nirala et al. [27]. Coal derived from wood has proved to be an excellent precursor for the synthesis of carbon nanomaterials as it guarantees an efficient electrochemical oxidative cleavage in multilayer graphene sheets. Electrochemical oxidation was performed in a two-electrode system by modulating the current intensity and the electrochemical cleavage was guaranteed both by the free radicals of the water and the peroxide of ammonium sulfate. Free radicals of water act like “scissors”, cutting carbonaceous macromolecules into multilayer sheets of graphene, and facilitate the easy insertion of SO_4_·radicals to easily insert into the sheets and, finally, reduce them to GQDs with peculiar structural and optical features. One of the disadvantages of the oxidative method is that in many cases it requires the use of strong acids or dangerous and harmful strong oxidants, i.e., non-eco-compatible approaches. Recently, the use of hydrogen peroxide as an oxidant, suggested by Lu et al., represents a valid green approach to obtain GQDs with good stability, applicable as fluorescent probes for bioimaging. Indeed, this latter process can be applied to several raw materials such as biomass wastes. Likewise, Liu et al. [35] used hydrogen peroxide in an environmentally friendly process for the synthesis of quantum dots from waste of the coke industry. Coal tar previously suspended in H_2_O_2_ was treated at 100 °C under reflux for 2 h, to obtain a monodispersed solid fluorescent GQDs (~1.7 ± 0.4 nm) with a high yield of more than 80 wt%. Similarly, Halder et al. [36] used hydrogen peroxide as a green oxidant to synthesize GQDs, starting from graphene oxide (GO) mixed with H_2_O_2_ (2–6%) at 180 °C for 2 h. The quantum dots obtained can be applied as fluorescent nanoprobes with high features for bioimaging, diagnostics, and drug delivery [37], since they showed high photostability and substantial biocompatibility as evidenced by cell viability tests. The method can be considered green, despite the fact that the pre-synthesized GO was obtained by treating synthetic graphite flakes with H_2_O_2_, concentrated sulfuric acid, concentrated hydrochloric acid, phosphorus pentoxide, potassium persulfate, potassium permanganate, and quinine sulphate. Using the same precursor but a different approach, Su et al. [38] synthesized nitrogen doped GQDs (N-GQDs) as promising probes for bio-imaging, from graphene oxide (GO), ethylenediamine and hydrogen peroxide. First, GO was synthesized from expandable graphite by the modified Hummers method [39] (Figure 2a) and then treated in an autoclave at 200 °C for 3 h. An interesting electrochemical exfoliation approach for the large-scale production of GQDs was proposed by He et al. [40], using coke obtained by pyrolysis at 1000 °C as starting material. The electrochemical process was carried out in a two-electrode system, with a piece of advanced coke as the reference electrode and a platinum plate as the counter electrode, using as electrolyte (NH_4_)_2_S_2_O_8_ dissolved in a mixture of MeOH and H_2_O. The authors found that the amount of water in the electrolyte solution and the applied current density affect the multi-color fluorescence and the size of the obtained GQDs, ranging from 3.02 to 4.61 nm with fluorescence emissions at 500, 530, and 560 nm, respectively. The reaction parameters also play an important role in the quantum yields allowing the production of high yields from 13.04to 42.86 wt% and 31.13 wt%, making possible eventual industrial scalability of the production process. Furthermore, GQDs were available for engineering of their solid-state GQDs/epoxy composites for advanced applications in multicolor light-emitting diode instruments. Finally, in a recent report of Duarte de-Menezes et al. [41], quantum dots were synthesized via a green electrochemical process based on the electrolysis of citric acid and sodium citrate for 24 h. Several tests of the mutagenicity in vivo of the nanoparticles performed on the obtained GQDs investigated their cytotoxicity and evidenced their possible use in human applications.

### 2.2. Laser Ablation

Chemical oxidation methods require the use of strong acids which can damage instruments in the long run and can be considered non-eco-friendly approaches. Therefore, simpler and more environmentally friendly alternatives have been developed such as pulsed laser ablation in the liquid phase. This technique was used by Narasimhan et al. [42] to set up fluorescent probes of GQDs for bio-imaging applications. GQDs were synthesized from a graphite plate immersed in an aqueous solution of polyethylene glycol submitted to laser ablation: a nanosecond pulsed laser source was optically directed in the direction of the substrate for 30 min causing the ablation of the graphite plate surface, obtaining GQDs in solution, subsequently separated by centrifugation and filtration, and larger graphene sheets settled to the bottom. The GQDs produced by this method showed good properties as fluorescent biomarkers. More recently, Kang et al. [43] employed pulsed laser ablation in the liquid phase, for the single-step synthesis of GQDs, from graphite flakes suspended in a solution of ethanol and 3-mercaptopropionic acid (MPA), pointing the pulsed laser source onto the suspension for 30 min at room temperature. The pulsed laser caused the decomposition of graphite flakes to form carbonaceous nanoparticles. C binds to S, derived from the decomposition of MPA, producing sulfur-doped graphene nanosheets. One of the main disadvantages of laser ablation is the high cost of the instruments, and until today only a few numbers of examples of GQD synthesized by laser ablation from eco-friendly raw materials have been reported.

### 2.3. Controllable Synthesis

The controllable synthesis process, over the years, has aroused less success because it includes complex chemical reactions in several phases, which require a long time [44]. The method starts from small molecules (derivatives of substituted benzene) to obtain quantum dots of colloidal graphene with the desired size and morphology [45]. A recent interesting report advanced by Lu et al. [46] consists of the controllable synthesis of GQDs with an average diameter of about 3.5 nm from glucose coupled with a hydrothermal treatment for 3 h at 200 °C. The main advantage of this technique is that, in addition to synthetic glucose, any natural source or biomass waste with high glucose content can easily be exploited as synthetic precursors. Previously, Naik et al. [47] produced GQDs from citric acid pyrolyzed for 25–30 min. The heating caused its decomposition and the formation of a hydronium ion acting as catalyst in the subsequent decomposition reactions, leading to the formation of GQDs from the nucleation of aromatic clusters. 1.5 M NaOH was added to the citric acid solution and the effects of pH on synthesis yield and UV-Vis absorption spectra were investigated. 

### 2.4. Pyrolysis 

Under severe reaction temperature and inert atmosphere, many organic molecules can be carbonized into graphitized materials for subsequent exfoliation of GQDs. Pyrolysis is one of the simplest carbonization processes and consists of exploiting high temperatures to convert various renewable starting materials into carbonaceous nanoparticles. A facile pyrolysis synthesis of monodisperse GQDs has been developed for the first time by Mahesh et al. [31], via emulsion-templated carbonization of carbohydrates from honey/water emulsion in the presence of butanol. The GQDs obtained from honey have been applied as transparent security ink and a component for white-light emission. Instead, Hassanzadeh et al. [48] used glucose directly for the synthesis of GQDs. They prepared a chemiluminescent biosensor to detect cholesterol using MoS_2_ nanosheets and graphene quantum dots synthesized by pyrolysis of glucose at 180 °C, for a few minutes. The obtained GQDs have an average diameter of 14.5 ± 4.6 nm and a maximum emission at an excitation wavelength of 360 nm, which gave a quantum yield of 46%. Veeramani et al. [49] synthesized porous graphene sheet-like carbon nanoparticles (GPACs) via carbonization from *Bougainvillea spectabilis* flowers. The flowers were crushed, dried, and subsequently pyrolyzed for 6 h at 200 °C. The synthesized material was exploited for the preparation of electrodes for the detection of catechin. Earlier, Kalita et al. [50] used rice grains as biomass by using a similar synthetic approach. In particular, the raw material was fried in a pan at 200 °C for different times, then dispersed in deionized water, and finally subjected to filtration. The high temperature causes the hydrolysis of the glycosidic bonds of the rice starch to decompose into glucose monomers, that subsequently undergo nucleation. The synthesis yield of GQDs from rice grains was approximately 56% and the obtained GQDs showed excellent fluorescence properties with the possibility of exploitation for the preparation of bio-imaging probes. A large scale and controllable synthesis of GQDs, consisting of a combination of two approaches, was recently proposed by Wang et al. [28]: the first belongs to the bottom-up approaches and involves the conversion of rice husk, and the second is a top-down approach, consisting of a hydrothermally assisted method. The rice husk was dried, crushed, and carbonized in a tubular stove in N_2_ atmosphere at 700 °C and reacted with NaOH at 900 °C for 2 h. The ash obtained was first suspended in an aqueous solution of sulfuric acid and subsequently solubilized by ultrasound for 5 h, then suspended again with the addition of nitric acid and ultrasonicated for 10 h. The solution was washed, filtered, and placed in an autoclave at 200 °C for 10 h. During this process, the silica contained in the rice husk was simultaneously used to synthesize mesoporous silica nanoparticles. This combined approach may open the door to new high-throughput syntheses of GQDs starting from new natural precursors.

### 2.5. Hydrothermal Method

As mentioned above, pyrolysis is the typical thermal method widely used for preparing nanoparticle carbonaceous materials starting from biomass. However, it has the disadvantage that the carbon source is progressively converted into dots via a series of heating, dehydration, degradation, and carbonization phenomena under drastic conditions requiring high temperatures and long reaction times. On the contrary, hydrothermal treatment, which consists of thermochemical degradation under mild conditions, by exploiting its high moisture content, could represent a valid top-down green technology for GQD synthesis from several natural feedstocks. In fact, the hydrothermal technique is today the most commonly used green technique, from simple precursors such as glucose, sucrose, citric acid and more complex materials, such as biomass wastes. A new interesting hydrothermal approach for converting biomass into GQDs was recently proposed by Wang et al. [51]. They synthesized GQDs, with an average size of ca. 3.9 nm with 2−3 graphene layers, by treating rice husk at 150 °C for 5 h in a Teflon-lined autoclave. As prepared, the RH-GQDs can be steadily dispersed in water, exhibiting intense photoluminescence and a highly selective quenching to Fe^3+^ ions, making them a promising material for Fe^3+^ ions sensing. Hydrazine hydrate assisted hydrothermal cutting, at 150/200 °C for 6–10 h, has been successfully applied by Wang et al. [9], for the synthesis of quantum dots starting from coffee grounds, resulting in blue luminescent GQDs. The obtained GQDs, after functionalization with polyethylenimine (PEI), showed enhanced fluorescent properties related to band-edge photoluminescence with single exponential decay, and their sensing and bioimaging applications were documented by Wang. An advancement in the hydrothermal approach was obtained by Wang et al. [52], who synthesized S-doped GQDs by hydrothermal carbonization of durian. The latter was crushed and dispersed in deionized water, then carbonized in an autoclave for 12 h at 150 °C, in the presence of platinum as a catalyst. It has been demonstrated that the low molecular weight saccharides contained in durian are the main sources of sp2 carbon. Furthermore, the high quantum yield and stable luminescence of the synthesized S-GQDs suggest that they can be successfully exploited in bio-imaging. Tade and Patil in a very recent study reported a simple approach for converting waste biomass of bamboo wood. The synthesis of GQDs was obtained by hydrothermal treatment at 180 °C for 8 h of cellulose nanocrystals (CNC), obtained from bamboo wood (Bf) and previously prepared [53]. The carbonaceous product was then subjected to ultrasound, subsequently filtered and finally lyophilized. The authors evidenced that the high temperatures and pressures required by hydrothermal treatment ensure the catalytic conversion of CNCs into one-pot GQD since the cleavage of the 1,4-glycosidic bond of cellulose, and the intramolecular polymerization leading to the formation of the graphite structure, occur almost simultaneously [54]. Indeed, the morphological and optical characterization of the synthesized Bf-GQDs encouraged their use as fluorescence sensors for the detection of curcumin. Ahmed et al. [55] used corn powder for the green synthesis of GQDs. The corn powder was dispersed in EtOH and stirred at 40 °C for 25 min and hydrothermally heated in an autoclave at 200 °C for 10 h. After one day of cooling, the product was solubilized in deionized water and then centrifuged seven times. The obtained multifunctional GQDs were efficiently used as an additive or as interlayers in perovskite solar cells (PSC) representing a new and effective way, in terms of environmental impact and economic feasibility, for PSC commercialization. Recently, Foong et al. [56] prepared several samples starting from sucrose, by varying the concentration of sucrose and solvent (mixture of EtOH and H_2_O). The sample was heated in an autoclave up to 190 °C for 12 h. The product obtained was dried in an oven at 80 °C for 24 h and then purified. The characterization confirmed the success of the synthesis from sucrose. Furthermore, the results showed that the addition of additives such as FeCl_3_ and oxalic acid significantly improve the quality of the final product. Bayat et al. [57] used glucose powder as a synthetic precursor of graphene quantum dots. Quantum dots of single-layer graphene were synthesized by hydrothermal treatment of the glucose powder in deionized water at 200 °C for 8 h. In a recent work of Chen et al. [58], polymeric cellulose was used as a synthetic precursor to obtain GQDs via an easy, eco-friendly, and one-pot hydrothermal approach. Previously, the same authors have synthesized GQDs from starch with a green method [59]. No acids or corrosive substances were used in this process, only water and cellulose. The cellulose is ultrasonicated for 30 min and the suspension obtained is subjected to HTC for 8 h at 180 °C involving a first step of hydroxylation followed by a phase of ring-closure condensation. The obtained GQDs show low cytotoxicity and good photoluminescence behavior with consequent high potential for in vitro cell imaging of human cervical carcinoma cells. Quantum dots of N-doped graphene modified with CuO/ZnO nanoarrays were synthesized by Safaei-Ghomi et al. [60] via a simple, green hydrothermal process for 9 h at 180 °C. Ethylenediamine and citric acid were dissolved in deionized water and mixed with the CuO/ZnO heterojunctions already prepared by a one-step hydrothermal process. Then, N-doped GQDs were formed by direct pyrolysis with 2-hydroxypropan-1,2,3-tricarboxylic acid and the hybrid composite generated with CuO/ZnO nanoarrays. In a recent approach, Zhu et al. [61] proposed the synthesis of GQDs by pyrolysis of citric acid, in a round bottom flask at 200 °C for 30 min. The GQDs were then functionalized with glycine, under alkaline conditions, at 120 °C (Gly-GQDs). The latter was characterized and the maximum fluorescence QY was obtained by optimizing the reaction at 120 °C for 90 min and pH 12. The Gly-GQDs were tested as highly effective fluorescence sensors for detection of Hg^2+^ in water because of the real quenching effect of metal ions by a non-radiative electron transfer. Again, by pyrolysis of citric acid, Hasanzadeh et al. [62] synthesized GQDs, used for the development of electrodes useful in detecting doxorubicin in the blood. Citric acid was pyrolyzed at 200 °C for 5 min until an orange liquid was obtained which was poured dropwise into an aqueous solution of NaOH. The solution thus obtained was characterized by showing a SEM graphitic morphological structure. The obtained sample was used for the preparation of electrodes, whose doxorubicin detection activity was studied by cyclic voltammetry. According to previous authors, Qu et al. [63] synthesized N-GQDs by HTC of citric acid solubilized in water and ammonia for 3 h at 200 °C. The product was then dialyzed for 8 h. The N-GQDs obtained were mixed with both tyrosinase (TYR) and acid phosphatase (ACP) and the relative fluorescence spectra were detected. The same composites were used in human blood samples, demonstrating the synthesis of a new fluorescence biosensor for detecting the activity of these enzymes. Xu et al. [64] used lignin sulfonates (SL), an uncommon biomass obtained from extraction of lignin from wood. Citric acid was firstly pyrolyzed by a green hydrothermal method at 200 °C for 15 min, transferred into a mixture of NaOH and lignin sulfonate (SL), and finally dialyzed in a bag for two days. A similar process was carried out but without the addition of SL dispersion and the obtained SL/GQD composites show excellent fluorescence revealing an emission intensity of the SL/GQDs four times higher than that of free GQDs. The peculiar features of the SL/GQDs have been exploited for the detection of Fe^3+^ (Figure 2b) and, to evaluate the specificity of the sensor, the PL intensities were measured in the presence of other interfering ions: the composite with Fe^3+^ showed the strongest fluorescence quenching efficiency. As emerged from the references reviewed, citric acid is a green precursor widely used in the synthesis of nanocarbon materials. Therefore, as previously mentioned for glucose, waste citrus fruits containing a high amount of citric acid could be exploited as interesting bio-precursors for the green synthesis of GQDs.

### 2.6. Microwave Irradiation Method

Microwaves are a form of electromagnetic radiation lying between infrared radiation and radio frequencies. Microwave heating is a simple, fast and economical process that is extensively used in the synthesis of different classes of materials, including GQDs [65]. Radiant energy is uniformly transferred to the substrate without direct interaction with the source, making the MW assisted approach more efficient than conventional heating. A one-pot microwave irradiation method was reported by Kumawat et al. [32] with mango leaves, exploited to synthesize GQDs, which were then used for in vivo imaging application. The mango leaves were minced, extracted in ethanol for 4 h, and mixed in water. The suspension was treated in the microwave for 5 min and, finally, centrifuged and filtered again. The obtained GQDs showed high biocompatibility and effectiveness for the detection of intracellular temperature. More recently, Abbas et al. [66] synthesized GQDs from tea waste by the microwave assisted oxidative process followed by a hydrothermal treatment in designing selective fluorescent sensors for the detection of the Fe^3+^ metal ion. The tea scraps were previously washed and dried for 12 h and, subsequently ground. Finally, they were subjected to pyrolysis in an oven at 500 °C for 3 h. The biochar obtained was subsequently subjected to oxidative cutting in a microwave reactor for 15–180 min. The material obtained was purified by hydrothermal treatment at 200 °C for 8 h. The prepared quantum dots showed high detection sensitivity. For the synthesis of quantum dots, plants have proved to be excellent precursors, thanks to their high carbon content. For this reason, the alcoholic extract of a climbing plant (*Clitoria ternatea*) was used by Tak et al. [67]. The *Clitoria ternatea* flower extract was mixed with HPLC water and subsequently heated to 900 W in a microwave oven for 5–10 min, then the resulting residue was distributed in absolute ethanol to form a GQD dispersion. The latter was filtered and the particles dried. The peculiar features of the GQDs obtained by this rapid synthesis were analyzed in vivo, evidencing the ability to significantly inhibit the enzyme acetylcholinesterase, and suggesting the possibility of exploiting GQDs in the treatment of Alzheimer’s disease. Dager et al. [33] have recently performed a synthesis of graphene nanoparticles using a one-step decomposition process, enhanced by microwave plasma. In a recent bottom-up method, Wu et al. [68] developed a synthetic alternative to GQDs for sensing applications by using microplasma, an innovative method in which the starting material is represented by fructose. Microplasma has already been used for the synthesis of semiconductor materials, electronic materials, or aerosols, but never for the synthesis of carbon-based quantum dots. This process does not find other sources in the bibliography, although the advantages of plasma treatment compared to other synthesis processes are already known: it increases the decomposition of the starting material, shortens the reaction time, and exploits the temperature produced during the reaction by not requiring an external power supply, therefore having excellent energy properties. The process takes place in a single step lasting 5 min. The pressure was monitored and kept constant and the internal temperature never exceeded 70 °C. A substrate holder, equipped with a halogen lamp heater, is placed under the plasma source. The carbon obtained is sonicated for 5 min, ultra-centrifuged for 10 min, and subsequently filtered. The yield of the described synthetic process was compared with that obtained with traditional synthetic methods, demonstrating its efficiency. Thakur et al. [69] proposed a microwave assisted heating one pot synthesis starting from pasteurized cow’s milk at different reaction times. The milk-derived multi-fluorescent GQDs, spherical in shape and with a lateral size of ca. 5 nm, were efficiently used in simultaneous bioimaging and drug delivery in cancer, using cysteamine hydrochloride as linker, demonstrating their possible use in drug delivery. GQDs functionalized with anti-cancer drug BHC using cysteamine hydrochloride as a linker molecule (GQDs@Cys-BHC) showed an 88% drug loading efficiency, and an in vitro drug release profile which was pH-responsive dependent. Moreover, GQDs have been demonstrated to be suitable for in vitro theranostic application in cancer therapy. By a similar approach, Li et al. [70] obtained GQDs O and S dual-doped (GCNQD) from citric acid and thiourea. The GCNQDs, with a luminescence behavior in the visible range highly dependent on the excitation wavelength and pH, denoting high fluorescence quantum yield (31.67%), strong resistance to the interference of high ionic strength environment, and good biocompatibility, were successfully used as fluorescent probes for HeLa cell imaging, suggesting a great potential in bioanalysis and related fields. Kumawat et al. [71] investigated a green method based on the use of alcoholic grape seed extract as a starting material. The extract was treated in a microwave after evaporation and dispersion in water obtaining GQDs that undergo “self-assembly” (sGQD) in the water, showing interesting cell proliferation activity in fibroblasts in vitro.

## 3. GQDs Obtained by Ecofriendly Synthesis for Electrochemical Sensors

Since the discovery of fullerenes in the late 1980s, several carbon nanomaterials have attracted the attention of researchers for the development of energy devices and as innovative electrode materials for environmental and electrochemical sensing applications, due to their large specific surface area and high electrical conductivity. One of the most valuable carbon- based products is graphene, offering a great potential for electrochemical and biosensing applications, because of its features such as ease and scalability of synthesis and subsequent functionalization, intense surface chemistry, and high biocompatibility [6,24,72,73]. However, the strong π–π bonding and van der Waals interactions, leading to a significantly reduced surface area due to the irreversible clustering of graphene sheets, hinder its real application possibilities [74]. QDs can be used as semiconductors; however, due to recombination and annihilation between electrons and holes, they suffer from the loss of electron conductivity and mobility, limiting their use [75]. On the other hand, GQDs, formed by breaking down the graphene into small dimension, non-zero band gap compounds, could act as semi-conductors. Furthermore, because of the enclosure and edge effects of quantum, GQDs denote higher speed electron transfers and conductivity, which, in addition to the respective optical features and strong luminescence, stand out as new materials combining the exceptional properties of both graphene and quantum dots [76]. In addition, they are soluble in water and can be easily functionalized through the hydrophilic groups present on their surface (e.g., hydroxyl, carboxyl, etc.), increasing their catalytic effect on various redox reactions [77]. Besides, GQDs of tunable sizes 2.2–0.3, 2.6–0.2, and 3–0.3 nm can be successfully used as multivalent redox species for the development of electrochemical sensors for the detection of heavy metals [78,79], small organic or inorganic molecules [80,81] and biological molecules [82,83,84,85,86].

In this section, the significant recent advances in the design of GQDs by eco-friendly synthetic routes [20,87], from biomass waste or other green raw materials, for the development of electrochemical sensors are summarized, with a comparative and balanced discussion (Table 2).

### 3.1. GQDs Electrochemical Sensors for Neurotransmitter Detection

Neurotransmitters are endogenous chemical messengers in the nervous system that influence a wide range of both psychological and physiological functions of the brain. Certain neurological hormones (dopamine, epinephrine, serotonin, etc.), hormone precursors (tyrosine, etc.) [34] and sex hormones (progesterone, ethinyl-estradiol, etc.) are essential in the correct functioning of the nervous, cardiac and muscular systems [88], and in controlling the correct functioning of the reproductive system [89]. Indeed, an abnormal level of their concentration in the human body leads to various neurodegenerative diseases such as Alzheimer’s, Parkinson’s, Huntington’s, depression, and schizophrenia [90]. For this reason, the use of fast, accurate, inexpensive, and biocompatible GQD electrochemical sensors that allow on-site detection, as well as the possibility of detecting two or more analytes instantaneously ensuring high selectivity and sensitivity, has aroused particular interest in scientific research. Hasanzadeh et al. [91] synthesized a nanocomposite of Fe_3_O_4_ magnetic nano-particles and graphene quantum dots, obtained by a sustainable pyrolysis of citric acid (Fe_3_O_4_MNP-GQDs), effectively suitable for the construction of a modified glassy carbon (GC) electrode, which exhibits an excellent electrocatalytic activity, at physiological pH, toward the electro-oxidation and detection of many amino acids such as L-cysteine, L-aspartic acid, L-phenylalanine and l-tyrosine. The GC-Fe_3_O_4_MNP-GQDs allowed the detection of L-Tyr in the linear ranges from 0.09–230.0 mM, showing an electrocatalytic activity much higher than that of individual GQDs and Fe_3_O_4_MNP, probably due to a synergistic effect of the high specific surface area and electrical conductivity of the nanocomposite, which facilitate the electron transfer process between the analyte and the electrode, also favoring electrochemical regeneration after the electronic exchange. Interesting results in the detection of L-Tyrosine (L-Tyr) have been obtained by Habibi and Heidari [92], using a carbon composite electrode modified with GQDs, obtained through citric acid pyrolysis, and subsequent deposition of RuCl_3_. The characteristics of the GQDs and the presence of RuCl_3_ resulted in a highly sensitive, stable and selective sensor, displaying detection limits of 0.23 μM and a sensitivity of 90 nA μM. Shadjou et al., on the other hand, produced a new polymeric nanocomposite sensor by incorporating GQDs, citric acid derived, into β-cyclodextrin (β-CD-GQDs) and subsequent electrodeposition on a glassy carbon electrode by CV. The nanocomposite modified electrode displayed high catalytic activity in the oxidation of L-Tyrosine in the linear ranges 0.1–1.5 μM, at physiological pH, due to a series of redox reactions between OH units and intermediate species by an electron transfer of L-Tyrosine [93]. GQDs, obtained by an analogous citric acid carbonization procedure, have been deposited by Li et al. on a glassy carbon (GC) electrode, to realize a self-assembled electrochemical device ((GQDs-NHCH_2_CH_2_NH)/GCE), revealing a high selectivity towards dopamine, determined by differential pulse voltammetry (DPV) analysis, and a sensitivity of 1306 μA mM^−1^ cm^−2^ and a LOD of 0.115 μM [94]. For the first time Ben Aoun, in 2017, presented a dopamine (DA) electrochemical sensor, obtained by the modification of a nanostructured screen printed carbon electrode (CSPE) with a chitosan/nitrogen doped GQDs nano composite, produced by a green microwave hydrothermal synthesis starting from glucose and chitosan (CS/N, GQDs@SPCE).

**Figure 3:**
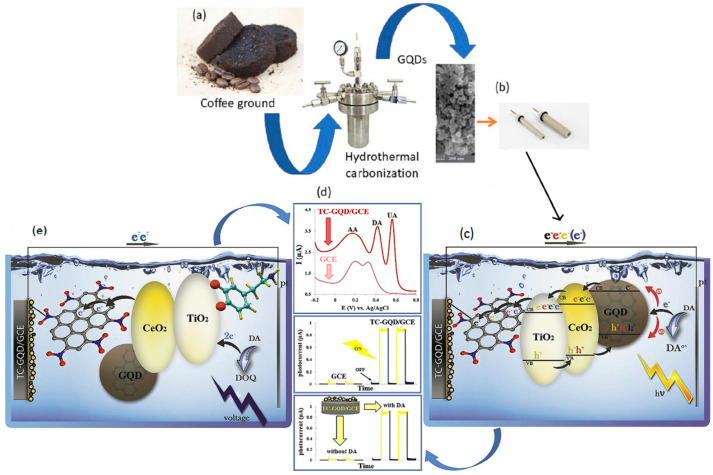
Electrochemical and photo electrochemical detection of dopamine on titania–ceria–graphene quantum dots nanocomposite. (**a**) GQD synthesis by hydrothermal carbonization process; (**b**) Fabrication of the EC and PEC electrodes by modification with the TC-GQD; (**c**) Schematic representation of the EC electron transfer mechanism at TC-GQD/GCE in the presence of DA; (**d**) Differential pulse voltammetry (DPV) of TC-GQD/GCE and photocurrent responses of the TC-GQD/GCE; (**e**) Schematic representation of the PEC electron transfer mechanism at TCGQD/GCE in the presence of DA. Adapted with the permission of Ref. [127].

The sensor showed high selectivity towards dopamine by DPV analysis, with linear ranges from 1 to 200 μM and very low LOD of 0.145 mM. In addition, chitosan provided high selectivity by broadening the dopamine potential peak relative to uric acid and blocking ascorbic acid interference, making the system suitable for the detection of dopamine in real samples of human urine [95]. Chitosan and GQDs, synthesized from citric acid, were also proposed by Tashkhourian et al. in 2018 to produce a new electrochemical sensor, doping a carbon paste electrode by electrochemical deposition. The aim of this study was to produce a voltametric sensor for the detection of epinephrine (EP). The GQD-CS-CPE sensor was tested by CV at a scan rate of 100 mV s^−1^ in the range of −0.30 to 0.90 V. Square Wave Voltammetry (SWV) analysis evidenced a linear range of 0.36 and 380 mM, without conflict with common interfering substances (ascorbic acid, dopamine and uric acid) [96]. An ultra-sensitive DA sensor was developed by Ruiyi et al. [97], involving histidine functionalization by the sol template method of a GQD-graphene micro aerogel from green citric acid pyrolysis. The electrochemical behavior of the obtained sensor (His-GQD-GMA), evaluated by means of differential pulse in the voltametric analysis (DPV), evidenced a good linear range for dopamine detection (0.001–80 μM) with LOD equal to 2.9 × 10^−10^ M at the S/N ratio equal to 3.144. Electrochemical sensors, highly active and selective for DA in human urine and serum real samples, have been obtained by the deposition of GQDs, produced by pyrolysis of biomass precursors, on carbon electrode together with ionic liquid. Square wave voltametric analysis was used for the determination of levodopa with a linear range of 0.05 to 250.0 μM. The voltametric study suggested that the oxidation of levodopa is significantly increased by the presence of GQDs and ionic liquid in the electrode. In addition, the RTIL-GQDs/CPE sensor was able to distinguish between the two signals for levodopa and serotonin [125]. In another study, a similar GQDs/IL-SPCE sensor provided a response by redox reaction with dopamine, ascorbic acid and uric acid. Cyclic voltametric analysis showed three well-defined signals for the simultaneous determination of the three substances. The linear ranges of dopamine, ascorbic acid and uric acid were 0.2–6 μM, 25–400 μM, 0.5–10 μM, respectively [126]. GQDs, synthesized via the bottom-up method of citric acid pyrolysis, were used by Zheng et al. [98] to modify a glassy carbon electrode. The large electroactive surface area, and the π–π bonding and electrostatic interaction between GQDs and dopamine, led to an excellent electrocatalytic activity in oxidizing dopamine at pH 7 and providing high sensitivity and selectivity. In a very recent approach, Ahmadi et al. used biomass-derived GQDs to modify a GCE to form a nanocomposite sensor doped with titania and ceria for electrochemical and photoelectrochemical determination of dopamine. The synthesis of GQDs was carried out by a green hydrothermal method from espresso coffee wastes, illustrated in Figure 3. These were subsequently reacted, by the same procedure, for the first time with titania-ceria nanocomposite. The TC-GQD electrode, investigated by cyclic voltammetry and differential pulse voltammetry analysis, allowed linear ranges for the electrochemical method of 1–500 μM and for the photoelectrochemical method of 0.3–750 μM [127]. Shiva Kumar Arumugasamy et al. [99] in 2020 produced an excellent sensor on glassy carbon modified with multiwalled functionalized carbon nanotubes (fMWCNTs) with GQDs synthesized from glucose. The GQDs@MWCNTs/GCE electrode was tested for the determination of dopamine showing good catalytic activity towards its oxidation. Cyclic voltametric analysis gave detection ranges of 0.25–250 μM with high selectivity, and good stability. One of the most interesting electrochemical approaches for the detection of dopamine and epinephrine in recent years was proposed by Vinoth et al.

The sensor was produced through the synthesis of GQDs using an ultrasonic method with a green substance such as glucose, then GQDs were deposited by ultrasonic irradiation onto the silicate matrix (TMSPED), previously treated with HAuCl_4_ × 3H_2_O to form the GQD-TMSPEDAuNCs electrode. The response ranges for dopamine (5 nM at 2.1 mM) and epinephrine (10 nM at 4.0 mM) were identified by amperometric analysis, and an excellent response in the simultaneous detection of the two neurotransmitters was highlighted [100]. Baluta et al. also produced a sensor in 2018 by modifying a glassy carbon electrode with GQDs (citric acid derivatives) for the detection of epinephrine. The GQDs were adsorbed on the electrode for 24 h forming a thin film. DPV analysis provided linear ranges of 1–120 mM. The laccase adoption in the electrode allows the measurement of low concentrations of epinephrine (>10 mM), negating the effect of interfering molecules [101]. Norepinephrine has been detected, by square wave stripping voltammetry analysis using GQDs derived from citric acid pyrolysis, modulating a glassy carbon electrode and a subsequent deposition of an AuNPs layer. The GCE/GQDs/AuNPs gave linear range values of 0.5–7.5 μM layer and no significant interference was detected [102]. In a recent approach, glassy carbon electrodes modified with GQDs (citric acid derivatives) doped with poly (sulfosalicylic acid) have been developed for simultaneous detection of estradiol (E) and progesterone (P) [103]. The same hormones have also been detected with good performance through CV analysis, by using an electrochemical sensor composed of GQDs, derived from citric acid pyrolysis, f-MWCNTs and Fe_3_O_4_ nanoparticles on a glassy carbon electrode (Fe_3_O_4_@GQD/fMWCNTs/GCE). Excellent sensitivity and selectivity results, probably due to a high degree of active sites favoring the oxidation of progesterone, were obtained with a linear range of 3.15–945 ng mL^−1^ [104].

### 3.2. Electrochemical Sensors for Biomarkers Detection

**Figure 4:**
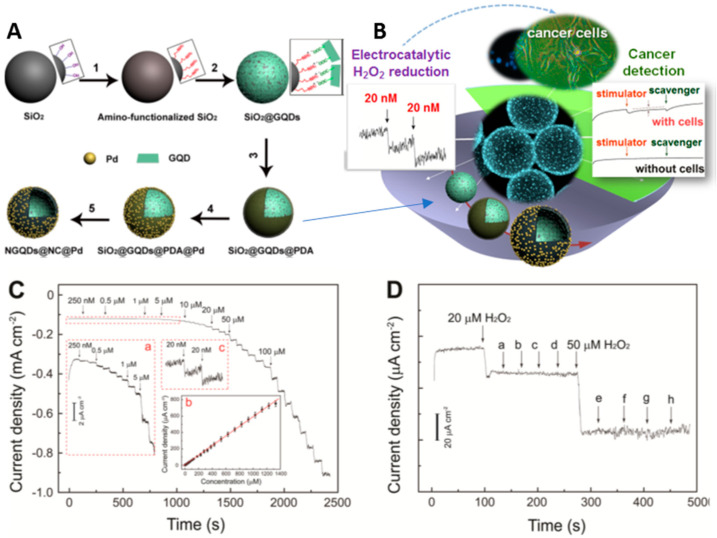
Preparation and application of N-doped graphene quantum dots@N-doped carbon@Pd hollow nanospheres in the H_2_O_2_ detection; (**A**) preparation process of NGQDs@NC@Pd HNSs; (**B**) schematic representation of the application field of GQD@NC@Pd/GCE sensor; (**C**) amperometric response of NGQDs@NC@Pd/GCE with successive step changes of H_2_O_2_ concentration; (**D**) influence of interfering compounds. Adapted with the permission of Ref. [105].

The relevance of electrochemical sensors for biomonitoring and biomarker analysis in environmental, industrial, agricultural, and above all clinical applications is increasingly being documented. In particular, in clinical scenarios, electrochemical sensing tools have been helpful in personalized medicine and minimally invasive diagnostic applications. Hydrogen peroxide (H_2_O_2_) can be considered one of the most frequent side products of reactions by oxidase in mitochondria, and can be used as a suitable biomarker for an early diagnosis of many types of cancer, since its adequate level in the human body plays a fundamental role in the control of living cells. As portrayed in Figure 4, Xi et al. proposed a useful amperometric sensor for the detection of hydrogen peroxide, using nitrogen-doped GQD (NGQD) grafted with Pd nanoparticles and encapsulated in carbon nanospheres to realize a hybrid material (NGQD@NC@Pd HNS). The combination of NCHNS, serving as electronically conductive catalyst support able to diminish ohmic resistance, and highly dispersed NGQD and Pd nanoparticles promote electron transfer as well as electro-catalytic activity for H_2_O_2_, resulting in a high-performance sensor, with a linear detection range up to 1.4 mM and low detection limit of 20 nM. Moreover, the GQD@NC@Pd/GCE sensor showed a high selectivity to H_2_O_2_, evidenced by anti-interference ability, without detectable amperometric responses in the presence of several electroactive interfering compounds, such as dopamine (DA), ascorbic acid (AA) and uric acid [105].

**Figure 5:**
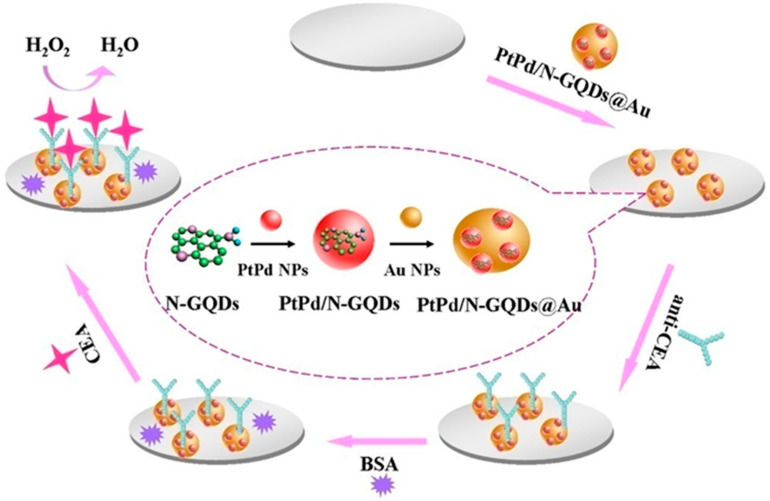
Schematic representation of the label-free electrochemical immunosensor and the preparation procedure of PtPd/N-GQDs@Au for the detection of carcinoembryonic antigen (CEA). Adapted with the permission of Ref. [107].

In another interesting approach, a non-enzymatic amperometric sensor has been developed for H_2_O_2_ detection by modifying a gold electrode with silver nano-cubes, chitosan and citric acid derived GQDs [106]. The Chit–GQDs/AgNCs electrode exhibited an excellent electro-catalytic activity in the reduction of H_2_O_2_, performing within a linear range of 10 mM to 7.8 mM and a LOD of 0.15 mM. Gold nanoparticles combined with Pd NPs, were also used to obtain, by electrodeposition of GQDs on a carbon fiber, an AuPd-ANPs/GQDs/ACF electrode with good H_2_O_2_ reduction skills, evidenced by sensitivity to H2O2 of 371 μAcm^−2^ mM^−1^ observed by means of cyclic voltammetry analysis [15]. In 2020, by the IR-assisted pyrolysis technique using citric acid and urea, a highly amidified GQD non-enzymatic and metal-free sensor was obtained. Amperometric analysis revealed excellent electrochemical response to H_2_O_2_, due to its reduction on the electrode, with a high amidation rate revealed by cyclic voltammetry studies. Authors observed that the GQDs electrode with a 1:1 urea/CA molar ratio exhibits the highest reductive current at a fixed rate, as compared to other samples, resulting in a noteworthy catalytic activity to H_2_O_2_ reduction. Indeed, the sensitivity and selectivity (1.83 μA mM^−1^ cm^−2^) of the sensor was excellent, highlighting its ability with small H_2_O_2_ concentrations [128]. A label-free sensing approach counting on antibody receptors was proposed by Yang et al. [107]. Nitrogen-doped GQDs, combined with Pt-Pd nanoparticles and Au, have been obtained via a multistep strategy, involving a first green hydrothermal carbonization of citric acid to form an N-GQD, further doped with Pd-Pt nanocomposite and the second step in gold nanoparticles deposition by a self-assembly process, to synthesize a PtPd/N-GQDs@Au hybrid. The schematic illustration of the label-free electrochemical immunosensor and the preparation procedure of PtPd/N-GQDs@Au.Y. is depicted in Figure 5.

The obtained nanocomposite, showing excellent chemical-physical features, such as high surface area, improved electron transferability, good biocompatibility and capability to catalyze H_2_O_2_, was used as a transducer to conjugate the capture of antibodies and to perform as a signal amplification structure. Indeed, the biosensor, fabricated by modifying a glassy carbon electrode (GCE) with PtPd/N-GQDs@Au, has been successfully investigated for the detection of carcinoembryonic antigen (CEA), exhibiting high specificity to CEA, with a linear range of 5 fg/mL to 50 ng/mL, LOD of 2 fg mL^−1^ and an insignificant response towards several interfering species such as BSA, IgG, PSA and hepatitis B surface antigen (HBS) [107]. The identification of organic molecules (e.g., drugs, vitamins, etc.) and DNA fragments (e.g., those derived from viruses) is also of particular importance for human health. Therefore, various electrochemical sensors have been developed for the detection of such molecules. Hasanzadeh et al., via a simple method of GQD deposition, synthesized by a bottom-up approach from citric acid on a glassy carbon electrode, managed to improve the performance of the CGE in detecting the drug, doxorubicin hydrochloride (DOX-HCl), through differential impulse voltammetry analysis. The CV profile advised that the coating of GQDs on GCE could enhance the performance in the electro-catalytic oxidation of DOX-HCl, while DPV conformed to the good DOX-HCl response in a wide linear range from 0.018 to 3.6 M, with an interesting LOD value of 0.016 M detected in human plasma [62]. In the same year, for the first time, the modification of a GCE was proposed with a novel nanocomposite obtained by electrodeposition β-cyclodextrin on GQDs, derived from citric acid, able to improve the redox reaction on the electrode surface. The so obtained β-CD-GQDs/GCE sensor was found effective in the detection of Vitamin C, evidencing a linear range of 0.01–170 μM determined by square-wave voltammetry analysis [108]. Very low concentrations of p-acetamido-phenol have been revealed by DPV analysis, using a nanohybrid of GQDs combined with proline (GNs/Pro-GQD), with a linear range of 0.08–100 μM and a tolerance limit towards interfering substances of less than 2.5% [129]. Li et al. in 2016 proposed a sensor for the detection of quercetin (Q), as a representative of the flavonoid family. The glassy carbon electrode was modified with gold nanoparticles and GQDs synthesized from black carbon obtained by using the wastes of burning candles. The electrochemical sensor showed great sensitivity and selectivity due to the excellent oxidation of the analyte, while DPV analyses provided linear ranges of 0.01 to 6.0 microM [109]. Samuei et al. in 2017, by means of the co-precipitation method, produced a non-enzymatic sensor with citric acid derived GQDs and CoNiAl-LDH nanocomposite. This was deposited on a carbon paste electrode and was used for glucose determination. Cyclic voltametric and amperometric analysis showed excellent electrochemical characteristics and high sensitivity of the sensor (48.717 μA mM^−1^) [130]. A label-free electrochemical platform for the highly sensitive detection of hepatitis B virus DNA, complementary to HBV-DNA bound to GQDs derived from citric acid, has been recently proposed. It was observed that the electrochemical signal, due to the action of electron-exchanging compound K_3_(Fe(CN)_6_), was slowed down by the strong binding to GQDs. However, when samples were tested with hepatitis B virus DNA, an increase in signal, proportional to the concentrations of the analyte was observed. Authors attributed this effect to the binding ability of probe DNA to HBV-DNA [110]. DNA analysis has also been performed by using a biosensor obtained by combining GQDs, produced by a green synthesis from citric acid, with polyvinylpyrrolidone-coated graphene oxide and gold nanoparticles. The electrochemical behavior of the obtained sensor was then investigated by amplified electro-chemiluminescent analysis, evidencing increased stability and signal enhancement with a linear range of 1.0 pM to 1000 nM and high sensitivity [111]. In a recent interesting approach, Trinadh et al. formed a sensor from GQDs, synthesized via a green approach from citric acid, doped with zirconium and lanthanum (GQDs@ La^3+^@ZrO_2_). The nanocomposite deposited on a glassy carbon electrode (GQDs@ La^3+^@ZrO_2_/GCE) was used for the detection of flutamide (FL), a cancer biomarker, in urine samples. The sensor showed a very good linear range (0.00175–15.75 µM) thanks to an excellent electrochemical response due to the drug reduction reaction. The presence of nanoparticles from the La^3+^@ZrO_2_ complex gave to the sensor an excellent stability and conductivity [112]. Arab et al. proposed a sensor for the electrochemical determination of paracetamol and 4-aminophenol. The sensor was produced by electro-polymerization of L-arginine on a glassy carbon electrode modified with GQDs, synthesized from glucose, functionalized multi-walled carbon nanotubes and a deep eutectic solvent (GQDs + DES + MWCNTs-COOH/PARG). The increased surface area available and the improved conductivity of the materials used improved the electrochemical behavior of the sensor, increasing the signal, due to the oxidation reaction of the analyte and allowing a linear range for the determination of paracetamol, determined by DPV analysis, of 0.10–110 mmol L^−1^ [131].

### 3.3. GQDs Based Electrochemical Sensors for Environmental Monitoring

**Figure 6:**
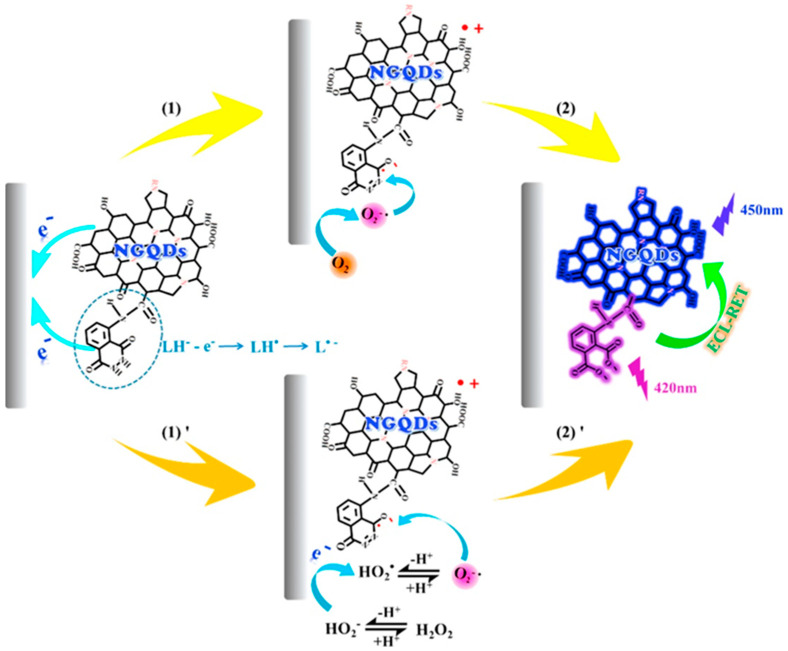
ECL-RET mechanism of luminol-NGQDs in H_2_O_2_ detection in water samples. Adapted with the permission of Ref. [119].

The significant development of industrial and agricultural activities greatly contributes to the improper discharge of pollutants into the environment. Consequently, environmental monitoring of toxic pollutants in various environmental media has become a great healthcare concern due to their lethal, hazardous, and adverse effects on both the environment and human health. Conventional methods employed for contaminant monitoring suffer from several drawbacks, requiring expensive devices and complex sample handlings, as well as being time-consuming and highly destructive. On this account, fast, simple and low-cost electrochemical sensing could represent a valid alternative strategy for pollutants monitoring in the environment [114,132]. Santos et al. in 2020 proposed GQDs, derived from citric acid, to modify a screen-printed electrode for the determination of ethinyl-estradiol (EE) in river water and human serum samples. The electrode was coated with a molecularly imprinted magnetic nanocomposite. The electrode response to ethinyl-estradiol detection was studied by voltametric analysis, providing a linear range from 1.0 × 10^−2^ to 2.5 μmol L^−1^. Moreover, electrochemical study of the electrode (mag@MIP)-GQDs-FG-NF/SPE) showed an increase in signal in the presence of ethinyl-estradiol without being affected by interference from other substances, allowing the use of the sensor for analysis on real samples [113]. Thus, in 2016 Xuan Jian et al. developed a modified glassy carbon electrode (GQDs/GCE) for the detection of hydroquinone (HY) and catechol (CA) in water, demonstrating how GQDs, synthesized from citric acid, could represent an excellent alternative for the construction of electrochemical sensors [114].GQDs/graphene/GCE has been successfully employed for the determination of Cu(II) in water, by differential pulse anodic voltametric analysis (DPASV), the authors inferring that combining GQDs with graphene allows a material able to enhance the electron movement, favoring the redox reaction on the electrode and showing a good sensitivity for copper (20.314 μA μM^−1^) and a good selectivity (error tolerance limit < ±5%) [115]. Moreover, modified GCE-GQDs poly(thionine) nanocomposites have been reported for the detection of Hg^2+^ ions [116]. Chun-Chieh Fu et al. in 2020, modified an indium-tin-oxide electrode with N-GQDs, synthesized from citric acid and urea, for the measurement of mercury ions. Electrochemical impedance spectroscopy analysis and cyclic voltammetry showed that the resistance decreases with increasing Hg^2+^ ion concentration, with a linear range of 0.05–0.25 μM. Indeed, the GQD/ITO electrode provided great selectivity with respect to the interferences tested and excellent sensitivity due to the high level of amidation and oxidation of the GQDs used [117]. A screen-printed electrode, doped with GQDs (citric acid derivatives) and aniline (1:4 *v*/*v* aniline monomer and GQDs solution ratio) for the detection of Cr(VI) in water, studied by means of linear sweep cathodic voltammetry, provided concentration limits for the determination of Cr(VI) of 0.1–10 mg L^−1^ with remarkable accuracy and great stability, making continuous sample detection possible(90 samples per hour) [118]. A sensor consisting of nitrogen-doped GQDs synthesized from citric acid and urea, and combined with luminol, has been recently investigated for the detection of hydrogen peroxide in water samples. Because of the doping of nitrogen atoms, NGQDs displayed good luminescence properties and, for the first time, a novel electrochemiluminescence resonance energy transfer (ECL-RET) process occurred between luminol, as the donor and NGQDs as the acceptor in the composite material. Indeed, the formation of the composite contributes to the two molecules approach, favoring the generation of RET, and then producing an anodic ECL signal without co-reactants. As evidenced in Figure 6, this results in an increased ECL signal of luminol-NGQDs during the electro-oxidation of hydrogen peroxide to O_2_^−•^ on the surface of electrode, highlighting the capabilities of the sensor in detecting H_2_O_2_ (linear range of 3.3 × 10^−8^~7.4 × 10^−5^ M) [119]. 

### 3.4. GQDs Based Electrochemical Sensors for Food Analysis

Environmental pollution also inevitably leads to food contamination by various molecules (heavy metals, toxins, pesticides, etc.). Since contamination usually has negative effects on food quality and can lead to a serious risk to human health, selective sensors have been developed for different types of contaminants. Through the pyrolysis of citric acid, Arvand et al. in 2016 synthesized GQDs for the development of an electrochemical sensor for the detection of L-DOPA in agricultural products. The GQDs were combined with FeCl_3_∙6H_2_O to form the magnetic compound Fe_3_O_4_@GQDs. This added to multi-walled carbon nanotubes was deposited on a glassy carbon electrode. The sensitivity and selectivity of the sensor were studied by differential pulse voltammetry showing a linear range of 3.0 to 400 µmol L^−1^ [120]. Wang et al. also, proposed a combination of GQDs, obtained in the same way, with Prussian blue and poly-pyrrole. The film was then deposited on a graphite electrode for the determination of L-cysteine in food and medical fields by CV analysis, showing a LOD of 0.15 microM and good selectivity towards interfering substances [121]. GQDs synthesized from D-(+)-glucosamine hydrochloride and poly(ethyleneimine), by microwave-assisted hydrothermal process, have been recently proposed for the preparation of a GQD and a molybdenum disulfide (MoS_2_) modified carbon-based screen-printed electrode for the detection of caffeic acid. The obtained nanocomposite proved to be a good support for binding the enzyme, able to improve the conductivity of the electrode. The detection of caffeic acid was investigated with good results by using cyclic voltammetry leading to a sensitivity of 17.92 nA μM^−1^ [133]. The presence of pesticides in the environment, naturally or as a result of human activity, has highlighted the need to develop sensors dedicated to their detection and measurement in food. Mehta et al. in 2017 presented the preparation of an electrochemical immunosensor based on GQDs (synthesized from citric acid) for the determination of parathion (PT). Through Electrochemical Impedance Spectroscopy analysis, a 46 pg/L detection limit was demonstrated. Moreover, the biosensor showed remarkable selectivity in the presence of the secondary metabolite parathion [7]. 

**Figure 7:**
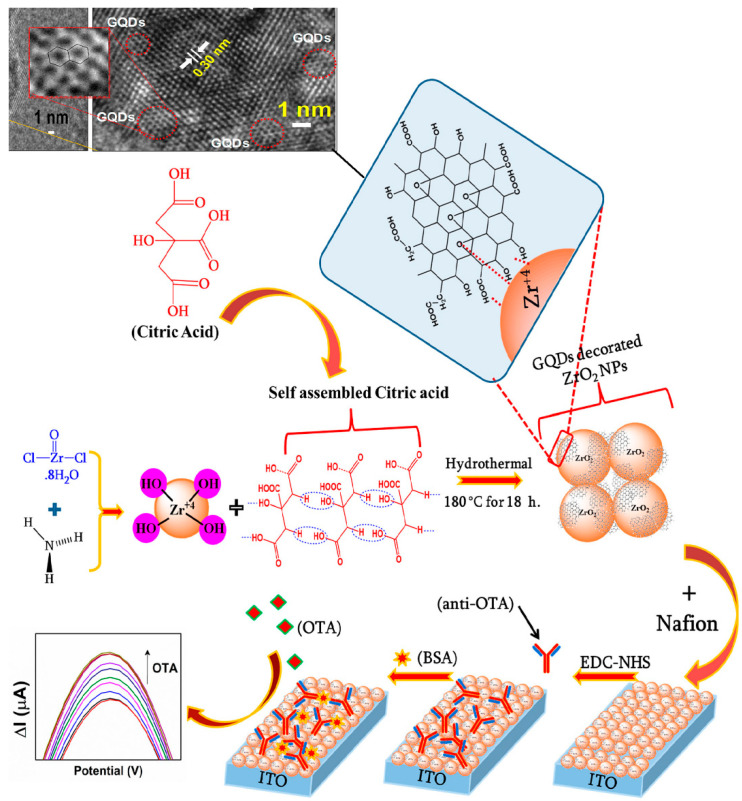
Detection of ochratoxin-A by electrochemical biosensing on (GQDs@ZrO_2_) electrodes. (Adapted with the permission of Ref. [124]).

Recently, Ghiasi et al. in 2020 developed an electrode for the organophosphorus pesticides (e.g., diazinon) detection. An activated glassy carbon electrode doped with GQDs (synthesized by citric acid pyrolysis), chitosan, and nickel molybdate nanoparticles (NMO/GQDs/CS/GCEox) coupled with a mini-tab software was used for the first time with the Taguchi method to find the best experimental conditions. By using DPV analysis, the sensor showed good selectivity and a linear range of 0.1–330 μM towards diazinon, with an increment of the current signal due to the amount of diazinon deposited on the electrode [122]. Detection of toxins that can contaminate various foodstuffs also plays an important role in food analyses. In 2018, Shadjou et al. proposed a new sensor for the determination of the M1 aflatoxin (AFM1), a vegetable food contaminant, e.g., in cereals. The electrochemical sensor was created through a multilayer film of GQDs, derived from citric acid pyrolysis, α-cyclodextrin, and silver deposited on a glassy carbon electrode (GQDs-α-CD-AgNPs). The study of the sensor response on the samples was performed by CV analysis demonstrating a linear range of 0.015 mM–25 mM [123]. Gupta et al. in 2020 presented a viable method for the detection of ochratoxin-A (OTA) by electrochemical biosensing. According to Figure 7, the biosensor was constructed with GQDs from citric acid, doped with zirconia nanoparticles (GQDs@ZrO_2_). A morphological study by TEM analysis clearly shows the existence of GQDs with lattice spacing values of 0.28 and 0.30 nm in the m(111) and t(101) planes of ZrO_2_ NPs. On their superficies, in fact, it is possible to see hexagons of carbon atoms of the GQDs. This system allowed electrons to move rapidly through the nanocomposite and prevented aggregation of GQDs by increasing the availability of their functional groups. This turns out to be relevant for antibody binding to the electrode. The BSA/anti-OTA/GQDs@ZrO_2_/ITO biosensor through CV and DPV analysis provided a sensitivity of 5.62 μA mL/ng cm^2^ and excellent stability and sensitivity toward ochratoxin-A [124].

## 4. Challenges and Future Perspectives

This review highlighted the last five years’ innovative research results on GQDs derived from renewable and green raw materials using eco-friendly approaches, and demonstrates their versatility for the manufacture of different sensing materials. Green synthesis derived GQDs are zero dimensional materials with a wide range of fascinating electrochemical properties. Due to their low dimension, they accomplish left-over benefits over their 2D counterparts. Indeed, synthetic carbonaceous nanomaterials represent a valid economic and biocompatible alternative to traditional carbon materials. This is challenging for the design of advanced electrochemical devices with high sensitivity. Furthermore, of particular interest are the composite materials that can be easily obtained through their functionalization. Finally, considering that one of the most attractive fields of application is their use as fluorescent tags on molecular targets in bioimaging, it appears clear that the tiny size of GQDs is a key benefit for further development of this diagnostic technique. We focused our attention on the most relevant progress related to GQD synthetic processes that find waste renewable substances and green materials of natural origin as precursors, and their application in the design of electrochemical sensors. In particular, in the last five years, there has been an increase in interest in the exploitation of biomass from which good yields of carbon nanomaterials have been obtained. Various biomass has been used or ecological methods advanced, but not all these processes can guarantee high yields or constant dimensions. Furthermore, GQDs have been successful in sensor research, thanks to properties such as quantum confinement and edge effect. Today many biomasses could be exploited as GQD precursors, that make up a source with excellent features still to be discovered. In spite of evolving recent improvements, research on biomass derived GQDs is still in its early stage and many drawbacks need to be overcome. Indeed, despite the fact that biomass waste could be a low cost and easily available raw material, the total cost of production might be higher compared to conventional unrenewable precursors. Furthermore, when it comes to detection, the field is still limited to the revelation of metal ions by exploiting the extinguishing properties of the fluorescence. Promising results were obtained by doping, but not all the properties of doped GQDs have yet been explained. However, it should be noted that, although in recent years research has widely focused on the use of waste biomass for the eco-compatible synthesis of GQDs, to achieve the standards imposed by green chemistry and with a view to sustainable development, very few significant examples of electrochemical sensors obtained with GQDs from biomass have yet been proposed. In this context, research based on the use of natural raw materials, such as citric acid and glucose, is much more prolific. Such precursors, although not real biomass, are still materials with a very low environmental impact, due to their easy extraction from agro-industrial products, including waste, and are very suitable for use as raw materials in green processes, without the use of harmful or toxic substances. From a future perspective, therefore, an improvement in synthesis methods is envisaged to increase understanding of both the morphological and dimensional characteristics of both applications from the materials obtained. GQDs represent an exciting future and the progress of nanotechnology will allow the development of highly selective and stable electrochemical sensors and biosensors obtained from easy and ecological synthetic processes. Finally, we hope this article will contribute to inspire further insights in this developing and fascinating research topic.

## Figures and Tables

**Table 1:** GQDs synthesized from different natural sources through green approaches by several techniques and their applications.

Source	Method	Application	Ref.
wood charcoal	Electrochemical oxidation	Detection of H_2_O_2_ and glucose	[27]
coal tar pitch	Chemical oxidation	Fluorescent probes	[35]
graphene oxide	Chemical oxidation	Fluorescent nano-probes	[36]
graphene oxide	HTC	Fluorescent probes for bio-imaging	[38]
coke	Electrochemical oxidation	Fluorescent properties for multicolor light-emitting diode devices	[40]
citric acid and sodium citrate	Electrochemical oxidation	Tests of the mutagenicity	[41]
graphite plate	Laser ablation	Fluorescent probes	[42]
graphite flakes	Laser ablation	Production of sulfur-doped graphene nanosheets	[43]
glucose	Controllable synthesis and HTC	Electrochemical luminescence devices	[46]
citric acid	Controllable synthesis	Determination of GQDs properties	[47]
honey	Pyrolysis	Biocompatible fluorescent ink	[31]
glucose	Pyrolysis	Chemiluminescent biosensor for the detection of cholesterol	[48]
Bougainvillea spectabilis flowers	Carbonization	Electrodes for detention of catechin	[49]
rice grains	Pyrolysis	Fluorescent properties	[50]
rice husk	Carbonization and HTC	Test for biocompatibility	[28]
coffee grounds	HTC	Bio-imaging	[9]
durian	HTC	Bio-imaging	[52]
bamboo wood	HTC	fluorescence sensors	[53]
corn powder	HTC	Solar cells	[55]
glucose powder	HTC	Determination of energy levels	[57]
cellulose	HTC	Cell imaging	[58]
citric acid	HTC	Detection of doxorubicin	[62]
sucrose	HTC	Photocatalytic activity	[56]
citric acid and ethylenediamine	HTC	QY of GQDs	[60]
citric acid	HTC	Fluorescent biosensor for TYR and ACP	[63]
citric acid	HTC	Fluorescent sensor of Hg^2+^	[61]
citric acid and lignin-sulfonate	HTC	Fluorescent sensors for Fe^3+^	[64]
tea waste	Microwave	Sensors for the detection of the Fe^3+^	[66]
mango leaves	Microwave	Detection of intracellular temperature	[32]
cow’s milk	Microwave	Drug delivery	[69]
citric acid and thiourea	Microwave	Fluorescent probes for bio-imaging	[70]
grape seed extract	Microwave	Photoluminescent Sensing Applications	[71]
fructose	Microplasma	Sensors for silver ions	[68]

**Table 2:** Electrochemical performance of eco-friendly GQD based sensors for the detection of several analytes.

GQDs Based Sensor	Detection Method	Analytes	Linear Range (μM)	LOD (μM)	Ref.
GQD-RuCl_3_/CCE	DPV	L-Tyr	1–937	0.23	[92]
β-CD-GQD/GCE	DPV	L-T	0.1–1.5	0.1	[93]
(GQDs-NHCH2CH2NH)/GCE	DPV	DA	1–150	0.115	[94]
CS/N,GQDs@SPCE	DPV	DA	1–200	0.145	[95]
GQD-CS-CPE	SWV AMP	EP	0.36–380	0.0003	[96]
His-GQD-GMA	DPV	DA	0.001–80	0.29 nM	[97]
GQDs/GCE	DPV	DA	0.4–100	0.05	[98]
GQDs@MWCNTs/GCE	CV	DA	0.25–250	0.095	[99]
GQD-TMSPED-AuNC	AMP	DA EP	0.005–2.1 0.01–4	0.005 0.01	[100]
GCE/GQDs/Lac	CV	EP	1–120	0.083	[101]
GCE/GQDs/AuNPs	SWSV	NEP	0.5–7.5	0.15	[102]
GQDs-PSSA/GO/GCE	DPV	E P	0.001–6 0.001–6	0.23 0.31	[103]
Fe3O4@GQD/fMWCNTs/GCE	DPV	P	0.0031–0.945	0.00063	[104]
NGQD@NC@Pd/GCE	AMP	H_2_O_2_	1400	0.02	[105]
Chit-GQDs/AgNCs/GE	AMP	H_2_O_2_	10–7380	0.15	[106]
PtPd/N-GQDs@Au	AMP	CEA-H_2_O_2_	5 × 10^−5^–0.05	2 × 10^−9^	[107]
β-CD-GQDs/GCE	SWV	AC	0.01–170	0.49	[108]
GQD/AuNP/GCE	DPV	Q	0.01–6.0	0.002	[109]
DNA/GQDs/GCE	CV, DPV	DNA	0.01–0.5	0.001	[110]
PDDA-GO/GQDs/DNA-gold NPs	ECL	DNA	1.0 × 10^−6^–1.0	1.0 × 10^−7^	[111]
GQDs@ La3 + @ZrO_2_/GCE	CV; EIS	FL	0.00175–15.75	0.00082	[112]
(mag@MIP)-GQDs-FG-NF/SPE	SWV	EE	10 × 10^−3^–2.5	0.0026	[113]
GQDs/GCE	DPV	HY CA	4.0–600 6–400	0.40 0.75	[114]
GQDs/graphene/GCE	DPASV	Cu^2+^	0.015–8.775	0.00134	[115]
GCE/PTH-afGQDs	CV	Hg^2+^	1 × 10^−6^–1	6 × 10^−7^	[116]
N-doped GQD/ITO	CV,EIS-	Hg^2+^	0.05–0.25	10 ppb	[117]
PANI/GQD-modified SPCE	LSV	Cr^6+^	100–1000	97	[118]
luminol-NGQDs	ECL	H_2_O_2_	0.033~74	0.01	[119]
(Fe_3_O_4_@GODs/f-MWCNTs/GCE	DPV	L-DOPA	3–400	14.3	[120]
PPy/GQDs@PB/GF	CVs-CA	L-cys	0.2–50 50–1000	0.15	[121]
NMO/GQDs/CS/GCEox	DPV	DZ	0.1–330	0.027	[122]
GQDs-α-CD-AgNPs-GCE	CV	AFM1	15–25,000	2	[123]
BSA/anti-OTA/GQDs@ZrO_2_/ITO	CV,DPV	OTA	1–20 ng/mL	0.38 ng/mL	[124]

## Data Availability

The data presented in this study are available in the article.
